# Creation of an Interactive Dashboard to Facilitate Early Detection of Cardiac Amyloidosis in African American Veterans

**DOI:** 10.1055/a-2513-9400

**Published:** 2025-05-14

**Authors:** Hamza Ghannam, Vikram Singh, Alberta L. Warner, Ariel Powell, Ramona Gelzer Bell, Kevin Chow, Kimberly D. Braswell, Rene Hearns, Vinod Aggarwal, Celina Roy, Douglas Stoehr, Jenice Guzman-Clark, Sandesh Dev

**Affiliations:** 1 University of Arizona College of Medicine, Tucson, Arizona, United States; 2 Department of Cardiology, VA Greater Los Angeles Healthcare System, David Geffen School of Medicine at UCLA, Los Angeles, California, United States; 3 Department of Cardiology, James A Haley Veterans' Hospital, Tampa, Florida, United States; 4 Department of Cardiology, VA Northeast Ohio Healthcare System, Cleveland, Ohio, United States; 5 VHA Office of Healthcare Innovation and Learning, VA Central Office, Washington, Dist. of Columbia, United States; 6 MDClone Limited, Be’er Sheva, Israel; 7 Department of Cardiology, Southern Arizona VA Health System, Tucson, Arizona, United States; 8 Department of Complex Adaptive Systems and Consulting, Arizona State University, Scottsdale, Arizona, United States

**Keywords:** dashboard, cardiac amyloidosis, electronic healthcare record, African American veterans

## Abstract

**Background** Cardiac amyloidosis (CA) is an underdiagnosed cause of heart failure (HF) that disproportionately impacts men of African descent. Without a standardized method of screening and scattered patient health information, clinicians must integrate data that spans multiple disease systems and is stored across the electronic health record.

**Objectives** The aim of this project was to create a dashboard to facilitate identification of high-risk African American (AA) veterans who would benefit from CA screening tests. This paper described the development of the dashboard and identified barriers and opportunities in dashboard development.

**Methods** Three Veterans Affairs (VA) health systems participated in this project. Microsoft Structured Query Language (SQL) Report Builder was utilized to create an interactive dashboard that refreshes daily through stored procedures using SQL Server Integration Services and the SQL Server Job Agent. Inclusion criteria included AA patients less than 90 years old with a history of HF. The 2023 American College of Cardiology/American Heart Association consensus statement on diagnosis and treatment of transthyretin CA was the source of evidence in creating the inclusion criteria and parameters of interest.

**Results** The final dashboard contained 1,732 HF patients who met inclusion criteria, of which 949 (55%) were identified as high risk. We faced several challenges in this project, including time required for dashboard development, limited team experience in specifying dashboard requirements, identifying informatics counterparts at all sites, and standardizing data across three VA hospitals.

**Conclusion** In this clinical improvement project, we created a dashboard that identifies AA veterans with HF at risk for CA and that can help to mitigate the impact of CA on this population.

## Background and Significance

It is important to identify the underlying disease etiologies that cause the syndrome of heart failure (HF), which impacts 6 million Americans. 1 Cardiac amyloidosis (CA) is a cardiomyopathy caused by the accumulation of transthyretin amyloid fibrils within the myocardium, or less often, by the accumulation of serum light chains. It was previously thought to be rare 2 but is now an increasingly recognized etiology of HF. 3 Although there is geographic variability, approximately 1% of unselected patients and 12% of HF patients with preserved ejection fraction have transthyretin (ATTR) CA. 4 In comparison to the general population, African American (AA) men of older age are disproportionately affected by CA because 3 to 4% of AA's are carriers of the V122I ATTR mutation, which causes hereditary ATTR. 5 In a study comparing the incidence of CA in 2012, AA men and women were twice as likely to have CA than Whites of the same sex. 6 This is particularly important for veterans because 16% of patients in the Department of Veterans Affairs (VA) health care system are AA, of which 85% are men. 7 There is an urgent need for more effective disease detection 8 because disease-modifying therapies have become available as of 2019. Disease detection has become easier due to the development of noninvasive diagnostic tests and the availability of CA screening recommendations in the 2022 HF guidelines. 9

However, CA remains underdiagnosed due to lack of standardized screening, the siloed nature of clinical information, 10 low clinician awareness, and a historical dependence on invasive myocardial biopsy. Screening for CA is comprised of bone scintigraphy and laboratory tests to exclude light chain amyloidosis. Patients additionally require cardiac imaging, biomarker tests, assessment for amyloid-associated comorbidities, and tissue biopsies. 11 Further, diagnosis of CA requires multidisciplinary coordination beyond cardiology. Hence, an electronic health record (EHR) dashboard that compiles risk factors for CA that can easily be reviewed by a clinician will enable a comprehensive view of patient presentation and facilitate identification of those patients who may benefit from further CA screening. Clinical dashboards are useful due to their ability to collect, summarize, and effectively present timely data for improving patient care. 12 Clinical dashboards have been used for management of various conditions such as hospital-acquired infections, 13 sickle cell disease, 14 predictive modeling of cystic fibrosis deterioration, 15 and trending of childhood obesity. 16

This paper describes a clinical dashboard used to identify AA veterans with risk factors for CA based on current guidelines 11 as part of a quality improvement initiative. We describe dashboard features that optimized its use in clinical practice, including a write-back feature and social determinants of health (SDoH) screening. The patients identified using this dashboard are at high risk for CA and will be proactively considered for disease-altering treatment earlier than they would have if we relied on individual patient-clinician decision making.

## Objectives

1. Describe the development and key characteristics of a dashboard to facilitate a cardiac amyloid early diagnosis program.
2. Identify barriers and opportunities in dashboard development.
3. Identify future clinical informatics opportunities for cardiac amyloidosis.

## Methods

### Setting and Dashboard Users

Three VA health systems participated in this project: Tucson VA (TUC), Greater Los Angeles VA (GLA), and Tampa VA (TPA). The dashboard was used by physicians ( *n* = 6), nurse practitioners ( *n* = 8), and residents/fellows ( *n* = 2) among the three sites.

### Dashboard Software Platform, Data Query, and Storage

**Fig. 1:**
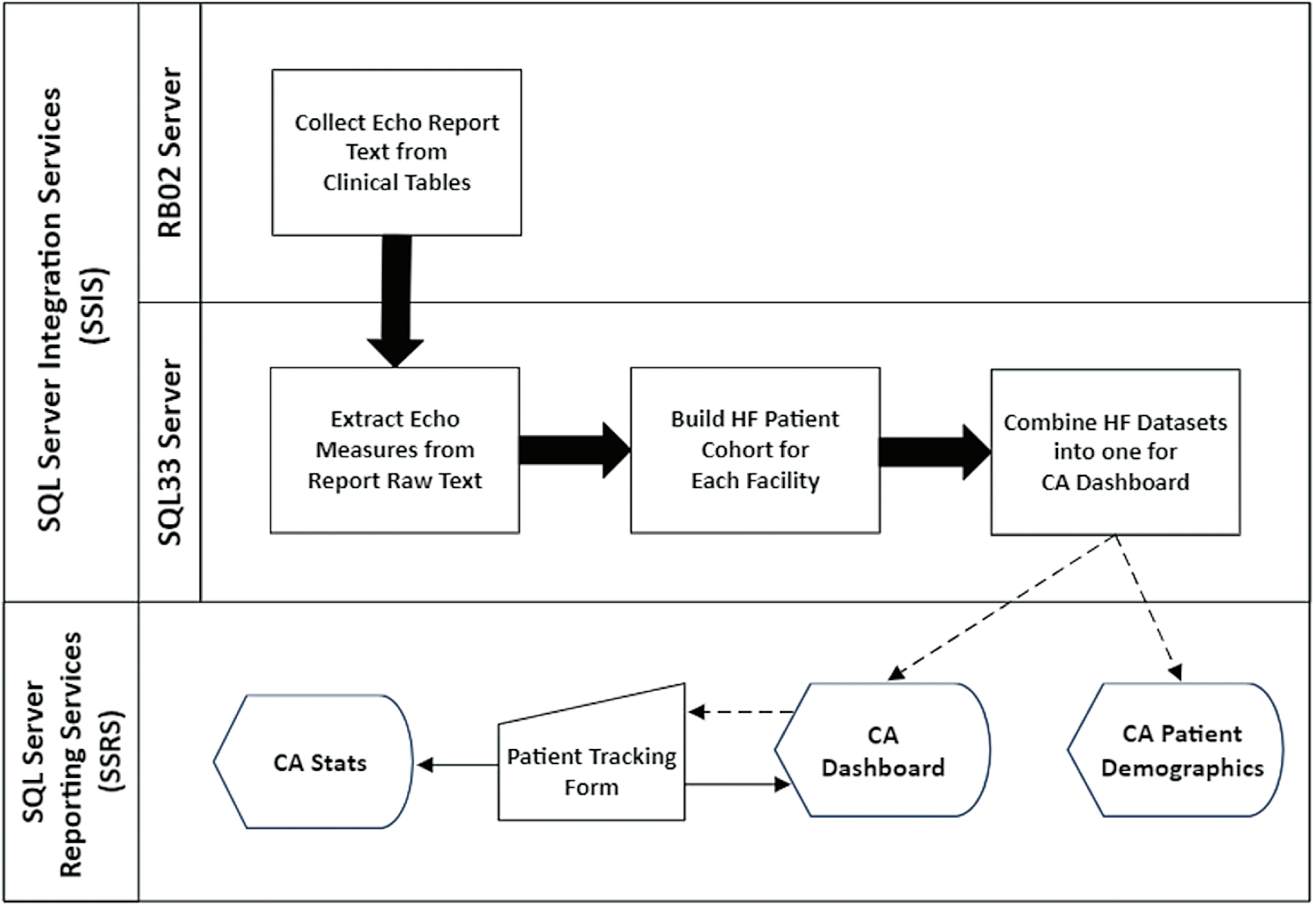
Diagram showing system architecture of our dashboard solution. CA, cardiac amyloidosis; echo, echocardiogram; HF, heart failure.

**Fig. 2:**
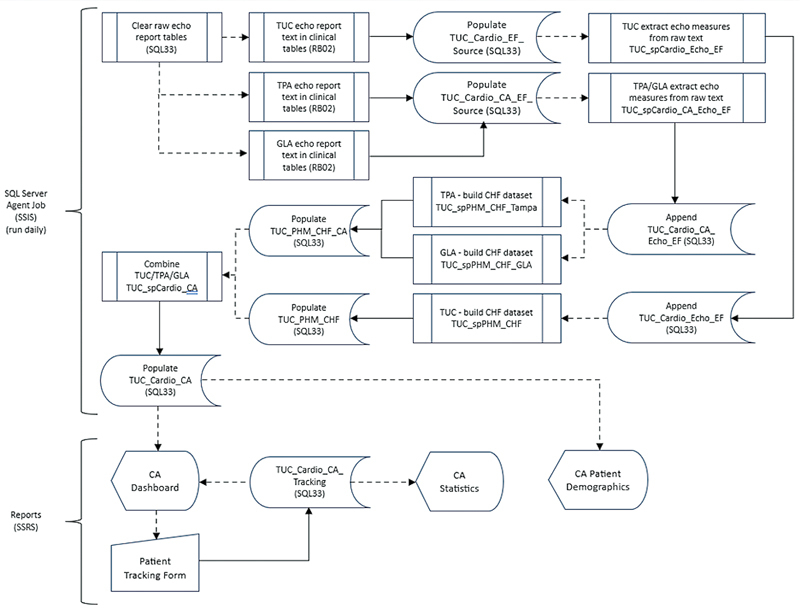
Unified modeling language diagram showing information flow in the various system components of our dashboard solution. echo, echocardiogram; CA, cardiac amyloidosis; CHF, congestive heart failure; GLA, Greater Los Angeles; sp, stored procedure; TPA, Tampa; TUC, Tucson. Solid line, movement of data; dashed line, reference to/source of.

The data source was the VA Corporate Data Warehouse (CDW), a central repository of all patient care-related data. Each VA facility has a local installation of the EHR—Veterans Health Information Systems and Technology Architecture (VistA)—which is then aggregated to the national VA CDW. The dataset is automatically refreshed daily through stored procedures using Structured Query Language (SQL) Server Integration Services and the SQL Server Job Agent. The dashboard was created using Microsoft SQL Report Builder (paginated report) for SQL Server Report Services. Several dashboard best practices were followed, including continuous clinician feedback regarding User Experience (UX) and User Interface (UI) to target visual appearance for optimized clinical use. 17 18 Figs. 1 and 2 depict a system architecture of our design with unified modeling language of data flow.

### Incorporation of Clinical Guidelines

**Fig. 3:**
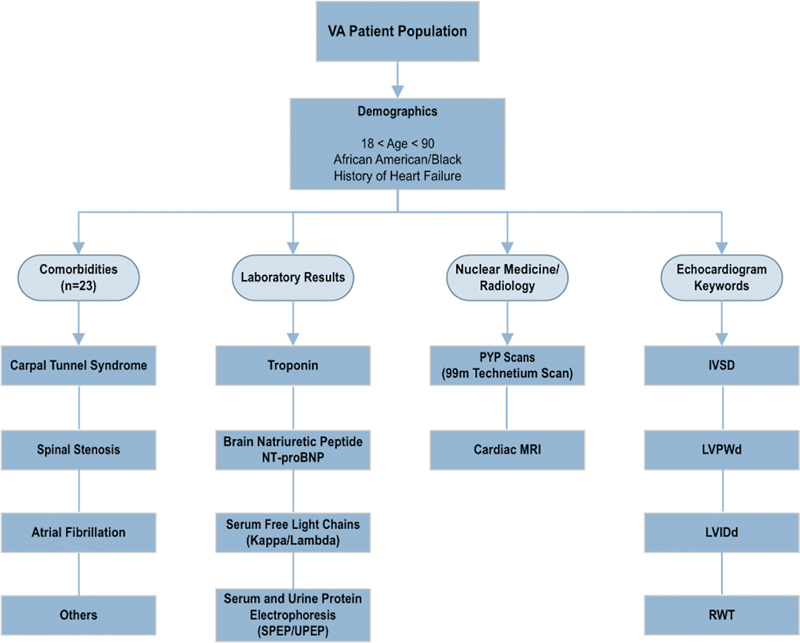
Summary of dashboard parameters. IVSd, interventricular septum thickness, diastolic; LVIDd, left ventricular internal diameter, end diastolic; LVPWd, left ventricular posterior wall thickness, diastolic; MRI, magnetic resonance imaging; RWT, relative wall thickness.

To identify the patient characteristics needed in the dashboard, we utilized the American College of Cardiology/American Heart Association (ACC/AHA) consensus statements 11 19 and clinical guidelines 9 on diagnosis and treatment of CA (see Supplementary Table S1 ). The clinical team reviewed the guidelines and applied their own clinical experience to specify the patient characteristics needed in the dashboard ( Fig. 3 ; for complete list: Supplementary Table S2 , available in the online version) to facilitate identification of at-risk patients.

### Dashboard Development

#### Inclusion and Exclusion Criteria

The target population included veterans that self-identified as Black/AA between the ages of 18 to 90 with a diagnosis of HF. HF was defined using International Classification of Diseases (ICD-10) codes and required a diagnosis listed in the patient problem list, ≥1 inpatient encounter for HF, or ≥2 outpatient encounters for HF within the past 2 years. Patients were excluded, if they had any of the following: existing amyloid (any type), relocated, current hospice, acute medical issues precluding further evaluation, history of medical noncompliance, or patient previously declining evaluation.

#### Demographics

**Fig. 4:**
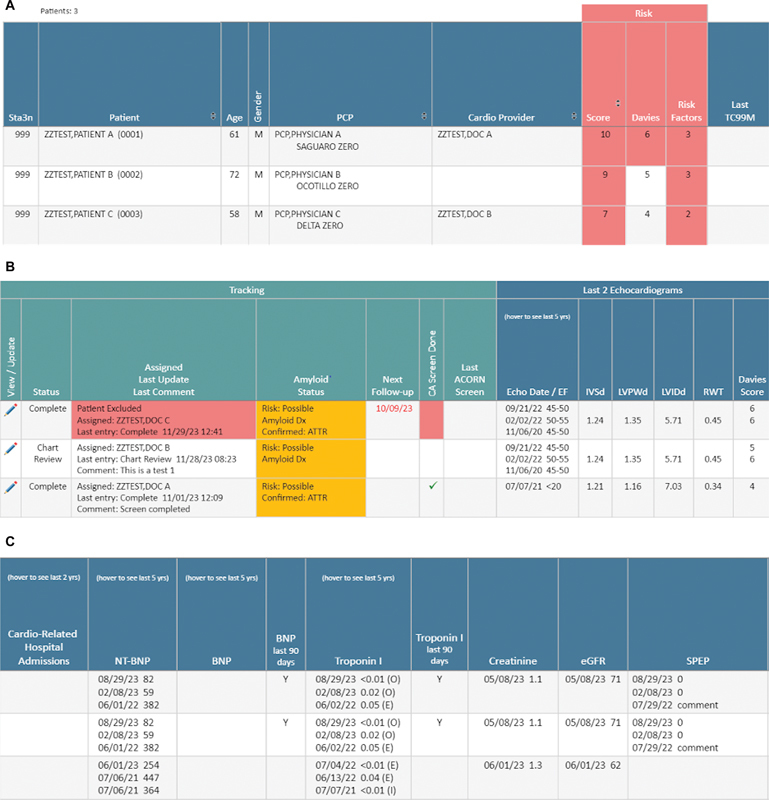
Sample screenshot of the dashboard. ( **A** – **C** ) Each row corresponds to an individual patient which displays horizontally across the dashboard screen. For presentation, we have partitioned the view of the dashboard into three separate panels ( **A** – **C** ).

The dashboard displays several aspects of patient information, including the patient's respective VA facility, name, age, assigned gender, body mass index, primary care provider, and cardiology provider ( Fig. 4A ).

#### Amyloid Risk Assessment Scores

There are two risk scores included in the dashboard. One is a published score comprised of age, sex, absence of hypertension, left ventricular (LV) posterior wall thickness, and LV relative wall thickness. 20 This score was developed based on retrospective analysis of patients at a tertiary care center. A score of ≥6 was associated with a 25% positive predictive value for ATTR CA. We used this best-available risk stratification score while being aware of its limitation that it lacked external validation. Further, the score was dependent on echocardiogram features and lacked assessment for amyloid-related diagnoses as recommended by practice guidelines. 19 To mimic optimal clinical practice, we developed a second risk score that consists of the sum of amyloid-related comorbidities ( Supplementary Table S2 , available in the online version) derived from the practice guidelines. 21 A score of ≥2 high-risk comorbidities was considered high risk based on team consensus.

#### Dashboard Output Parameters

Based on practice guidelines, 19 we included amyloid and nonamyloid-related comorbidities, laboratory tests, imaging studies, and medications that would be relevant to identify HF patients with possible amyloidosis ( Supplementary Table S2 , available in the online version). These output parameters were defined by ICD-10 codes ( Supplementary Table S3 , available in the online version).

Patients with known amyloid were excluded from additional review. Nonamyloid-related comorbidities that are commonly assessed by cardiologists were included. In particular, history of myocardial infarction was relevant, because recent infarction may lead to false-positive bone scintigraphy results. 11 History of cocaine and methamphetamine use was included as this might impact eligibility and adherence to treatment. The laboratory tests shown are routinely ordered in the evaluation of cardiac amyloid and HF. From echocardiogram semistructured text reports, we extracted key features using natural language processing (NLP; Supplementary Table S4 , available in the online version). We included the date of the last technetium-99m (PYP) scan or cardiac MRI for ease of finding the report in the EHR. Unfortunately, because electrocardiography images are not available in the CDW, we were not able to include these results. Amyloid-specific therapies were included as these may help clinicians identify patients already diagnosed with CA and exclude them from further review. We also included hydroxychloroquine, which can lead to false-positive bone scintigraphy results. 22

### Dashboard Risk Assessment and Tracking of Diagnostic Testing

**Fig. 5:**
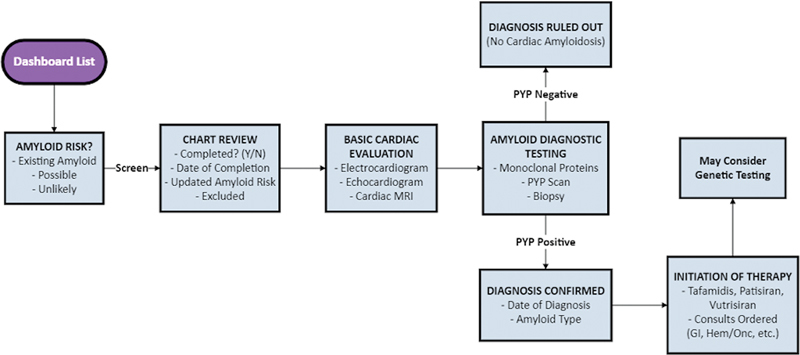
Workflow of screening patients for cardiac amyloidosis.

**Fig. 6:**
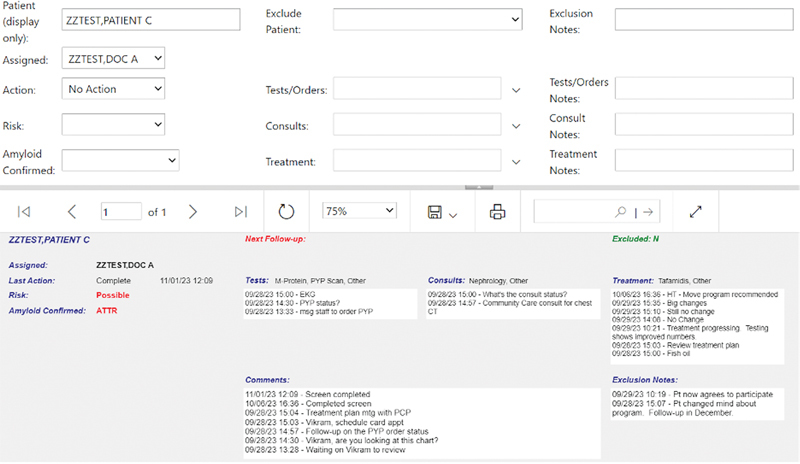
Screenshot of the writeback feature.

The clinical teams were instructed to review patient characteristics in the dashboard and the EHR in a standardized workflow manner ( Fig. 5 ) to decide if patients had sufficient risk to warrant additional diagnostic testing. To assist with tracking, we created a “write-back” feature to document clinical decision-making and facilitate downstream tracking of patient flow through the screening process ( Fig. 6 ).

### Social Determinants of Health Screening

For patients who underwent amyloid testing and subsequent evaluation in clinic, clinicians were asked to conduct screening for SDoH using an 11-question VA-specific questionnaire regarding social needs that may impact health, termed Assessing Circumstances and Offering Resources for Needs (ACORN) survey. 23 If this screening tool was completed, the results were available to the clinician in the dashboard.

### Summary Results and Outcome Visualization Function

**Fig. 7:**
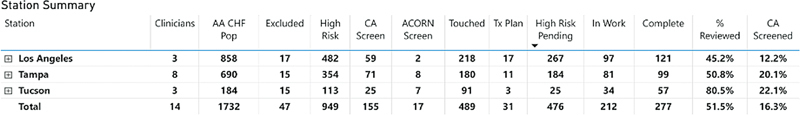
An outcome visualization function depicting the number of patients at each VA facility and their progress through the screening process by clinician. VA, Veteran Affairs.

We created an outcome visualization function page to allow for review of progress through the stages of diagnosis at each facility ( Fig. 7 ). Additional summary pages displayed averages or counts of demographics and comorbidities by facility.

The overall patient sample size in this initiative was 1,732 patients. There were 858 AA HF patients identified in GLA, 690 in TPA, and 184 in TUC, of which 949 (55%) were identified as high-risk for CA. A separate report will describe the outcomes and results of these patients identified by the dashboard.

### Data Validation

To ensure validity of the dashboard, clinicians manually cross checked the patient-specific dashboard information against the patient's EHR. Any data fields that were inconsistent or incomplete were reported. Errors were remedied by data analysts and additional features were developed based on clinical team consensus. This process was repeated over 12 weeks until the dashboard was deemed accurate and representative of the patient's EHR.

## Lessons Learned

This article describes the development of an interactive dashboard to facilitate early detection of CA in AA veterans with HF. This dashboard consolidates a myriad of features including risk scores, comorbidities, biomarkers, and imaging to facilitate a diagnosis of this complex disease. Aggregation of multiple data types in a simple user interface aimed to facilitate clinicians' ability to make rapid inferences as experienced by other authors. 24 The dashboard serves as an informatics tool that allows us to operationalize a proactive population health approach in a high-risk population.

Our incorporation of novel risk scores is an example of both the benefits and challenges of implementing research into clinical practice. We incorporated a validated risk score that was recently published 20 to guide clinicians in identifying HF patients who would likely benefit from further diagnostic testing. In the original publication, the score was validated in two external population samples, and therefore, we felt it was appropriate for use in our project. However, we did not validate its performance at our three sites prior to embarking on this project; this would have been impractical. It is important to note that our intent was to accelerate the translation of the risk scores into practice. Nonetheless, we will report on the effectiveness of this screening initiative which will help us to validate our approach. This project evaluation is concordant with the concept of the learning health system in which health systems utilize, as well as generate, new evidence in the process of patient care.

The use of NLP methods was critical in this project and highlights the need to incorporate multiple technical approaches to dashboard development. We extracted echocardiogram measurements to calculate CA risk score and targeted keywords from the echocardiogram reports that indicated higher risk of CA ( Supplementary Table S4 , available in the online version). Because the echocardiogram data was semistructured, we implemented NLP/regular expressions to extract these parameters. Other studies have employed NLP in research settings to identify combinations of cardiac/noncardiac phenotypes to predict transthyretin cardiomyopathy with good performance. 25 Given the amount of unstructured data in the EHR, we found that integrating NLP technology into the diagnostic framework for HF patients with less common etiologies is a promising direction.

Moreover, the challenges encountered during dashboard development are important to understand. Dashboard development was a lengthy process prior to project launch, with refinements occurring over the next 6 months. Team members had limited experience in specifying dashboard requirements that were translatable to data analysts. Another challenge was that data formats were not standardized across VA sites, and diagnostic test naming was frequently different. With the collaboration of data analysts and clinicians across the three sites, we were able to harmonize this after several dashboard revisions. However, as we scale this dashboard solution across 120+ VA medical centers we anticipate further challenges of data harmonization that will constrain the goal of data monetization.

There are strengths to our project as well. We had a clear purpose, understood the user, and knew what was being evaluated and how. These factors were critical for a successful design as has also been noted by Hysong et al. 26 The dashboard was functional and adequate to support clinician review after several months of development. It was also comprehensive and included most elements needed for risk-stratifying patients for CA. The write-back feature allowed users to capture decision-making and record risk assessments. The goal of automatic data collection with a clinician-familiar display of data to facilitate quick clinical decision-making was achieved. Nonetheless, further refinement and implementation efforts are likely needed to optimize usability and sustainment.

To our knowledge, this report is one of the earliest descriptions of a dashboard for CA. A study by Willis et al 27 sought to develop EHR alerts and other tools to identify HF patients at risk of CA by implementing combinations of phenotypes associated with confirmed cases of disease. However, this was never implemented into the EHR. In contrast, our dashboard was created to support immediate implementation of a clinical program for population screening based on current evidence and guidelines. We acknowledge that our dashboard-driven screening project could lead to unintended consequences, including unnecessary testing for CA-negative patients, technology overload leading to clinician burnout, and automation bias with overreliance on technology. We would like to acknowledge that clinicians are inherently limited by available knowledge of the risk factors of the disease. In our forthcoming evaluation of this project, we will report the effectiveness of screening for CA including false-positive tests.

There are several areas for future work. For our self-developed risk score, we would like to go beyond clinician guidelines and create a predictive model that utilizes unstructured and structured data features to identify ATTR CA in our patient population. 28 29 We plan to create EHR-embedded clinical decision support alerts, which would improve clinician workflow and increase the reach of this initiative. Because amyloidosis involves a multidisciplinary evaluation, this dashboard could be tailored to support workflows in related disciplines, such as hematology and neurology, to identify patients with light chain amyloidosis and transthyretin polyneuropathy, respectively. Finally, we would also like to incorporate additional data sources such as electrocardiography, echocardiogram, genetic testing, and other records, which will require additional data integration and processing techniques. Our dissemination efforts will initially focus on VA facilities, but this work can be replicated at non-VA health systems as well. However, as this project is a pilot initiative, we don't have a formal implementation plan yet.

## Conclusion

In this project, we created a dashboard to facilitate early diagnosis of CA among AA veterans with HF. This dashboard supports cardiologists and related disciplines by bringing together disparate clinical information from the EHR and provides patient-level risk stratification to facilitate early diagnosis. We anticipate that this tool will facilitate efforts to reduce underdiagnosis of CA and reduce unnecessary variation in care that may adversely affect patients including those from at-risk and underserved communities.

## Clinical Relevance Statement

Diagnosis and treatment of CA is an increasingly important area of cardiology practice with implications for several other specialties involved in care of these patients. This project demonstrates the value of a patient-level dashboard to facilitate early identification of cases and downstream care tracking. Given the numerous cardiac and noncardiac features of CA and complexity of diagnosis, this dashboard may serve as a template for other health systems that seek to employ a population approach to management of this underrecognized condition.

## Multiple-Choice Questions

1. Which of the following challenges is mentioned in the article regarding screening for cardiac amyloidosis?
  - Lack of diagnostic tools to diagnose the disease
  - Low prevalence to raise awareness
  - Complex disease process requiring a multidisciplinary approach
  - Irrelevant to diagnose given lack of medical therapy
  **Correct Answer** : The correct answer is option c. Because amyloid deposition affects multiple organs of the body, not just the heart, it is often missed unless a clinician, or group of clinicians, make the connection between several comorbidities. It is also a complex disease process that requires further research and understanding, making it a challenge to effectively screen and diagnose.
2. What types of patients does the dashboard specifically target for risk assessment?
  - Women under 75 years of age
  - All veterans regardless of race
  - Patients with confirmed heart failure, regardless of subtype
  - African American veterans with heart failure
  **Correct Answer** : The correct answer is option d. Cardiac amyloidosis often disproportionately affects African American men of older age. In a study comparing the incidence of CA in 2012, African American men and women were 2× as likely to have CA than their racial counterparts. Targeting veterans is especially important, as approximately 16% of the Veteran Health Administration is of African American descent.
3. What technology was used to extract echocardiogram report data for the dashboard?
  - Natural language processing
  - Machine-based learning
  - Manual entry by clinicians
  - Data queries from the Corporate Data Warehouse
  **Correct Answer** : The correct answer is option a. This was especially critical to the successful creation of this dashboard because echocardiogram measurements are essential to calculating a patient's ATTR risk score. Echocardiogram data are semistructured, meaning we had to utilize natural language processing and regular expressions to successfully extract these parameters.

## References

[1] Roger V L Epidemiology of heart failure: a contemporary perspective Circ Res 2021 128 10 1421 1434 33983838 10.1161/CIRCRESAHA.121.318172

[2] Rapezzi C Lorenzini M Longhi S Cardiac amyloidosis: the great pretender Heart Fail Rev 2015 20 02 117 124 25758359 10.1007/s10741-015-9480-0

[3] Martinez-Naharro A Hawkins P N Fontana M Cardiac amyloidosis Clin Med (Lond) 2018 18 02 s30 s35 29700090 10.7861/clinmedicine.18-2s-s30 PMC6334035

[4] Aimo A Merlo M Porcari A Redefining the epidemiology of cardiac amyloidosis. A systematic review and meta-analysis of screening studies Eur J Heart Fail 2022 24 12 2342 2351 35509173 10.1002/ejhf.2532 PMC10084346

[5] Chandrashekar P Alhuneafat L Mannello M Prevalence and outcomes of p.Val142Ile *TTR* amyloidosis cardiomyopathy: a systematic review Circ Genom Precis Med 2021 14 05 e003356 34461737 10.1161/CIRCGEN.121.003356 PMC8530896

[6] Gilstrap L G Dominici F Wang Y Epidemiology of cardiac amyloidosis-associated heart failure hospitalizations among fee-for-service Medicare beneficiaries in the United States Circ Heart Fail 2019 12 06 e005407 31170802 10.1161/CIRCHEARTFAILURE.118.005407 PMC6557425

[7] Washington D LJL Kasom D R Canning M National Veteran Health Equity Report - Black or African American Veteran Chartbook. Focus on Veterans Health Administration Patient Experience and Health Care QualityVHA Off Health Equity2023

[8] ATTR-ACT Study Investigators Maurer M S Schwartz J H Gundapaneni B Tafamidis treatment for patients with transthyretin amyloid cardiomyopathy N Engl J Med 2018 379 11 1007 1016 30145929 10.1056/NEJMoa1805689

[9] ACC/AHA Joint Committee Members Heidenreich P A Bozkurt B Aguilar D 2022 AHA/ACC/HFSA guideline for the management of heart failure: a report of the American College of Cardiology/American Heart Association joint committee on clinical practice guidelines Circulation 2022 145 18 e895 e1032 35363499 10.1161/CIR.0000000000001063

[10] Kiourtis A Mavrogiorgou A Mavrogiorgos K Electronic health records at people's hands across Europe: the InteropEHRate protocols Stud Health Technol Inform 2022 299 145 150 36325855 10.3233/SHTI220973

[11] Writing Committee Kittleson M M Ruberg F L Ambardekar A V 2023 ACC expert consensus decision pathway on comprehensive multidisciplinary care for the patient with cardiac amyloidosis: a report of the American College of Cardiology solution set oversight committee J Am Coll Cardiol 2023 81 11 1076 1126 36697326 10.1016/j.jacc.2022.11.022

[12] Ghazisaeidi M Safdari R Torabi M Mirzaee M Farzi J Goodini A Development of performance dashboards in healthcare sector: key practical issues Acta Inform Med 2015 23 05 317 321 26635442 10.5455/aim.2015.23.317-321 PMC4639357

[13] Makic M BF Stevens K R Gritz R M Dashboard design to identify and balance competing risk of multiple hospital-acquired conditions Appl Clin Inform 2022 13 03 621 631 35675838 10.1055/s-0042-1749598 PMC9179234

[14] Miller R Coyne E Crowgey E L Implementation of a learning healthcare system for sickle cell disease JAMIA Open 2020 3 03 349 359 33215070 10.1093/jamiaopen/ooaa024 PMC7660956

[15] Szczesniak R D Brokamp C Su W Mcphail G L Pestian J Clancy J P Improving detection of rapid cystic fibrosis disease progression-early translation of a predictive algorithm into a point-of-care tool IEEE J Transl Eng Health Med 2018 7 2.800108E6 10.1109/JTEHM.2018.2878534 PMC6368437 30800534

[16] Rescic N Alberts J Altenburg T M SmartCHANGE: AI-based long-term health risk evaluation for driving behaviour change strategies in children and youthInt Conference Applied Mathematics Comp Sci2023

[17] Kiourtis A Mavrogiorgou A Zafeiropoulos N Mavrogiorgos K Karabetian A Kyriazis D UI/UX sustainable design: best practices for applications co2 emissions reduction2024 9th International Conference on Smart and Sustainable Technologies (SpliTech).2024

[18] Rahmawati N Suroto S Syah T Kurniawan T Ramadani R Hotel administration application design using Delphi and UI/UX designer Internet Things Artificial Intelligence J 2024 4 03 395 405

[19] American Heart Association Heart Failure and Transplantation Committee of the Council on Clinical Cardiology Kittleson M M Maurer M S Ambardekar A V Cardiac amyloidosis: evolving diagnosis and management: a scientific statement from the American Heart Association Circulation 2020 142 01 e7 e22 32476490 10.1161/CIR.0000000000000792

[20] Davies D R Redfield M M Scott C G A simple score to identify increased risk of transthyretin amyloid cardiomyopathy in heart failure with preserved ejection fraction JAMA Cardiol 2022 7 10 1036 1044 36069809 10.1001/jamacardio.2022.1781 PMC9453635

[21] Writing Committee Members ACC/AHA Joint Committee Members 2022 AHA/ACC/HFSA guideline for the management of heart failure J Card Fail 2022 28 05 e1 e167 10.1016/j.cardfail.2022.02.010 35378257

[22] Chang I CY Bois J P Bois M C Maleszewski J J Johnson G B Grogan M Hydroxychloroquine-mediated cardiotoxicity with a false-positive ^99m^ technetium-labeled pyrophosphate scan for transthyretin-related cardiac amyloidosis Circ Cardiovasc Imaging 2018 11 01 e007059 29288196 10.1161/CIRCIMAGING.117.007059

[23] Russell L E Cohen A J Chrzas S Implementing a social needs screening and referral program among veterans: assessing circumstances & offering resources for needs (ACORN) J Gen Intern Med 2023 38 13 2906 2913 37165261 10.1007/s11606-023-08181-9 PMC10171907

[24] Shenvi E Boxwala A Sittig D Visualization of patient-generated health data: a scoping review of dashboard designs Appl Clin Inform 2023 14 05 913 922 37704021 10.1055/a-2174-7820 PMC10665122

[25] Moya A Oeste C L Beles M Detection of transthyretin amyloid cardiomyopathy by automated data extraction from electronic health records ESC Heart Fail 2023 10 06 3483 3492 37726928 10.1002/ehf2.14517 PMC10682883

[26] Hysong S J Yang C Wong J Knox M K O'Mahen P Petersen L A Beyond information design: designing health care dashboards for evidence-driven decision-making Appl Clin Inform 2023 14 03 465 469 37015343 10.1055/a-2068-6699 PMC10266903

[27] Willis C Watanabe A H Hughes J Applying diagnosis support systems in electronic health records to identify wild-type transthyretin amyloid cardiomyopathy risk Future Cardiol 2022 18 05 367 376 35098741 10.2217/fca-2021-0122

[28] Grogan M Lopez-Jimenez F Cohen-Shelly M Artificial intelligence-enhanced electrocardiogram for the early detection of cardiac amyloidosis Mayo Clin Proc 2021 96 11 2768 2778 34218880 10.1016/j.mayocp.2021.04.023

[29] Duffy G Cheng P P Yuan N High-throughput precision phenotyping of left ventricular hypertrophy with cardiovascular deep learning JAMA Cardiol 2022 7 04 386 395 35195663 10.1001/jamacardio.2021.6059 PMC9008505

