# Supplementary material for: Creation of an Interactive Dashboard to Facilitate Early Detection of Cardiac Amyloidosis in African American Veterans
Source: Appl Clin Inform. 2025 May 14;16(2):429–38. doi: 10.1055/a-2513-9400 (PMC12077983; doi:10.1055/a-2513-9400)
Supplement: Supplementary file 1 — Supplementary Material [file 10-1055-a-2513-9400-s202407ra0233.pdf]

Supplementary Table S1 Relevant source of information used in dashboard creation

Relevant source	Description	Rationale for implementation
ACC Consensus Guidelines (Kittleson[11])	Expert-derived consensus statement on diagnosis and treatment of cardiac amyloidosis	• List of amyloid associated comorbidities and features<br>• Description of diagnostic protocol
Simple score to identify increased risk of transthyretin amyloid cardiomyopathy in heart failure with preserved ejection fraction (Davies[20])	Novel score for identifying patients at high risk for cardiac amyloidosis	• Published risk stratification score to improve sensitivity and specificity of screening<br>• List of necessary echocardiogram measurements to identify high risk patients
Assessing circumstances and offering resources for needs (Russell[23])	Social determinants of health screening tool developed for use in veterans	• Part of project scope given known social barriers in veterans

Supplementary Table S2 Output parameters of dashboard

Amyloid-related comorbidities	Nonamyloid-related comorbidities	Laboratory tests	Imaging Studies	Medications
Atrial fibrillation/flutter	COPD	BNP/NT-proBNP	Echocardiogram	Diuretics
Other arrhythmias	Diabetes mellitus	Troponin I	Bone scintigraphy	ACE-I
Acute coronary syndrome	Diabetic neuropathy	HS Troponin I	MRI	ARB
Dysautonomia	Coronary artery disease	Serum creatinine		ARNI
Acute pericarditis	ESRD	eGFR		Beta blocker
Nephrotic syndrome	Current dialysis	SPEP		MRA
Syncope	Hx of cocaine Use	UPEP		SGLT2i
Hx of ICD	Hx of meth use	Free light chains		Tafamidis
Permanent pacemaker	Hx of Myocardial Infarction			Patisiran
Heart block				Diflunisal
Hx of TAVR				Hydroxy-chloroquine
Carpal tunnel				
Spinal stenosis				
Knee/hip prosthesis				
Hx of falls				
Biceps tendon rupture				
MGUS				

Abbreviations: ACE-I, angiotensin-converting enzyme inhibitors; ARB, angiotensin II receptor blockers; ARNI, angiotensin receptor neprilysin inhibitors; BNP, brain natriuretic peptide; COPD, chronic obstructive pulmonary disease; eGFR, estimated glomerular filtration rate; ESRD, end-stage renal disease; HS, high sensitivity; ICD, implantable cardioverter defibrillator; MGUS, monoclonal gammopathy of undetermined significance; MRA, mineralocorticoid receptor antagonists; MRI, magnetic resonance imaging; SGLT2i, sodium-glucose cotransporter-2 inhibitors; SPEP, serum protein electrophoresis; TAVR, transcatheter aortic valve replacement; UPEP, urine protein electrophoresis.

 © 2025. The Author(s).

Supplementary Table S3 ICD-9/10 codes with data definitions of dashboard

Data point	Definition
Patient cohort	Black or African American
	Congestive heart failure (see "patient diagnosis history")
	Age <90 y
	No hospice flag
Patient diagnosis history	ICD-10 code from problem list, inpatient, outpatient (LIP Dx only) past 2 y
	Qualifying Dx: PL or Inpt or 2+ Outpt
Congestive heart failure (AHRQ Definition)	I09.81,I11.0,I13.0,I13.2,I50.1,I50.20,I50.21,I50.22,I50.23,I50.30,I50.31,I50.32,I50.33, I50.40,I50.41, I50.42,I50.43,I50.810,I50.811,I50.812,I50.813,I50.814,I50.82,I50.83, I50.84,I50.89,I50.9
Hypertension (HTN)	I10.,I11.0,I11.9,I12.0,I12.9,I13.0,I13.10,I13.11,I13.2,I15.0,I15.1,I15.2,I15.8,I15.9, I16.0,I16.1,I16.9
Coronary artery disease (CAD)	I25.10,I25.110,I25.111,I25.112,I25.118,I25.119,I25.2,I25.3,I25.41,I25.42,I25.5,I25.6, I25.700,I25.701, I25.702,I25.708,I25.709,I25.710,I25.711,I25.712,I25.718,I25.719, I25.720,I25.721,I25.722,I25.728, I25.729,I25.730,I25.731,I25.732,I25.738,I25.739, I25.750,I25.751,I25.752,I25.758,I25.759,I25.760, I25.761,I25.762,I25.768,I25.769, I25.790,I25.791,I25.792,I25.798,I25.799,I25.810,I25.811,I25.812, I25.82,I25.83, I25.84,I25.89,I25.9
Chronic obstructive pulmonary disease (COPD)	J41.0,J41.1,J41.8,J42.,J43.0,J43.1,J43.2,J43.8,J43.9,J44.0,J44.1,J44.9,J47.0,J47.1,J47.9
Diabetes mellitus Type I, Type II (DMI, DMII)	E08.00,E08.01,E08.10,E08.11,E08.21,E08.22,E08.29,E08.311,E08.319,E08.321, E08.3211,E08.3212, E08.3213,E08.3219,E08.329,E08.3291,E08.3292,E08.3293, E08.3299,E08.331,E08.3311,E08.3312, E08.3313,E08.3319,E08.339,E08.3391, E08.3392,E08.3393,E08.3399,E08.341,E08.3411,E08.3412, E08.3413,E08.3419, E08.349,E08.3491,E08.3492,E08.3493,E08.3499,E08.351,E08.3511,E08.3512, E08.3513,E08.3519,E08.3521,E08.3522,E08.3523,E08.3529,E08.3531,E08.3532, E08.3533,E08.3539, E08.3541,E08.3542,E08.3543,E08.3549,E08.3551,E08.3552, E08.3553,E08.3559,E08.359,E08.3591, E08.3592,E08.3593,E08.3599,E08.36, E08.37 × 1,E08.37 × 2,E08.37 × 3,E08.37 × 9,E08.39,E08.40,E08.41, E08.42,E08.43, E08.44,E08.49,E08.51,E08.52,E08.59,E08.610,E08.618,E08.620,E08.621,E08.622, E08.628,E08.630,E08.638,E08.641,E08.649,E08.65,E08.69,E08.8,E08.9,E09.00,E09.01, E09.10,E09.11, E09.21,E09.22,E09.29,E09.311,E09.319,E09.321,E09.3211,E09.3212, E09.3213,E09.3219,E09.329, E09.3291,E09.3292,E09.3293,E09.3299,E09.331, E09.3311,E09.3312,E09.3313,E09.3319,E09.339, E09.3391,E09.3392,E09.3393, E09.3399,E09.341,E09.3411,E09.3412,E09.3413,E09.3419,E09.349, E09.3491, E09.3492,E09.3493,E09.3499,E09.351,E09.3511,E09.3512,E09.3513,E09.3519, E09.3521, E09.3522,E09.3523,E09.3529,E09.3531,E09.3532,E09.3533,E09.3539, E09.3541,E09.3542,E09.3543, E09.3549,E09.3551,E09.3552,E09.3553,E09.3559, E09.359,E09.3591,E09.3592,E09.3593,E09.3599, E09.36,E09.37 × 1,E09.37 × 2, E09.37 × 3,E09.37 × 9,E09.39,E09.40,E09.41,E09.42,E09.43,E09.44,E09.49, E09.51, E09.52,E09.59,E09.610,E09.618,E09.620,E09.621,E09.622,E09.628,E09.630,E09.638, E09.641, E09.649,E09.65,E09.69,E09.8,E09.9,E10.10,E10.11,E10.21,E10.22,E10.29, E10.311,E10.319,E10.321, E10.3211,E10.3212,E10.3213,E10.3219,E10.329,E10.3291, E10.3292,E10.3293,E10.3299,E10.331, E10.3311,E10.3312,E10.3313,E10.3319, E10.339,E10.3391,E10.3392,E10.3393,E10.3399,E10.341, E10.3411,E10.3412, E10.3413,E10.3419,E10.349,E10.3491,E10.3492,E10.3493,E10.3499,E10.351, E10.3511,E10.3512,E10.3513,E10.3519,E10.3521,E10.3522,E10.3523,E10.3529, E10.3531,E10.3532, E10.3533,E10.3539,E10.3541,E10.3542,E10.3543,E10.3549, E10.3551,E10.3552,E10.3553,E10.3559, E10.359,E10.3591,E10.3592,E10.3593, E10.3599,E10.36,E10.37 × 1,E10.37 × 2,E10.37 × 3,E10.37 × 9, E10.39,E10.40,E10.41, E10.42,E10.43,E10.44,E10.49,E10.51,E10.52,E10.59,E10.610,E10.618,E10.620, E10.621,E10.622,E10.628,E10.630,E10.638,E10.641,E10.649,E10.65,E10.69,E10.8, E10.9,E11.00, E11.01,E11.10,E11.11,E11.21,E11.22,E11.29,E11.311,E11.319,E11.321, E11.3211,E11.3212,E11.3213, E11.3219,E11.329,E11.3291,E11.3292,E11.3293, E11.3299,E11.331,E11.3311,E11.3312,E11.3313, E11.3319,E11.339,E11.3391, E11.3392,E11.3393,E11.3399,E11.341,E11.3411,E11.3412,E11.3413, E11.3419, E11.349,E11.3491,E11.3492,E11.3493,E11.3499,E11.351,E11.3511,E11.3512, E11.3513, E11.3519,E11.3521,E11.3522,E11.3523,E11.3529,E11.3531,E11.3532, E11.3533,E11.3539,E11.3541, E11.3542,E11.3543,E11.3549,E11.3551,E11.3552,

 © 2025. The Author(s).

Supplementary Table S3 (*Continued*)

Data point	Definition
	E11.3553,E11.3559,E11.359,E11.3591,E11.3592, E11.3593,E11.3599,E11.36, E11.37 × 1,E11.37 × 2,E11.37 × 3,E11.37 × 9,E11.39,E11.40,E11.41,E11.42, E11.43, E11.44,E11.49,E11.51,E11.52,E11.59,E11.610,E11.618,E11.620,E11.621,E11.622, E11.628, E11.630,E11.638,E11.641,E11.649,E11.65,E11.69,E11.8,E11.9,E13.00, E13.01,E13.10,E13.11,E13.21, E13.22,E13.29,E13.311,E13.319,E13.321,E13.3211, E13.3212,E13.3213,E13.3219,E13.329,E13.3291, E13.3292,E13.3293,E13.3299, E13.331,E13.3311,E13.3312,E13.3313,E13.3319,E13.339,E13.3391, E13.3392, E13.3393,E13.3399,E13.341,E13.3411,E13.3412,E13.3413,E13.3419,E13.349, E13.3491, E13.3492,E13.3493,E13.3499,E13.351,E13.3511,E13.3512,E13.3513, E13.3519,E13.3521,E13.3522, E13.3523,E13.3529,E13.3531,E13.3532,E13.3533, E13.3539,E13.3541,E13.3542,E13.3543,E13.3549, E13.3551,E13.3552,E13.3553, E13.3559,E13.359,E13.3591,E13.3592,E13.3593,E13.3599,E13.36, E13.37 × 1, E13.37 × 2,E13.37 × 3,E13.37 × 9,E13.39,E13.40,E13.41,E13.42,E13.43,E13.44,E13.49, E13.51, E13.52,E13.59,E13.610,E13.618,E13.620,E13.621,E13.622,E13.628,E13.630, E13.638,E13.641, E13.649,E13.65,E13.69,E13.8,E13.9
Diabetic neuropathy	E08.40,E08.41,E08.42,E08.43,E08.49,E08.610,E11.40,E11.41,E11.42,E11.43,E11.49, E11.610,E13.40, E13.41,E13.42,E13.43,E13.49,E13.610,G60.0,G60.1,G60.2,G60.3, G60.8,G60.9
Body mass index (BMI)	E66.01,E66.09,E66.3,E66.8,E66.9,Z68.30,Z68.31,Z68.32,Z68.33,Z68.34,Z68.35, Z68.36,Z68.37,Z68.38, Z68.39,Z68.42,Z68.43,Z68.44,Z68.45
Dialysis	I95.3,Z99.2
Implantable cardiac defibrillator (ICD)	Z45.02,Z95.810
Heart block	I44.0,I44.1,I44.2,I44.30,I44.39,I44.4,I44.5,I44.60,I44.69,I44.7
Permanent pacemaker (PPM)	Z45.010,Z45.018,Z95.0
Valve replacement (VR)	Z95.2,Z95.3,Z95.4
Atrial fibrillation (AFib)	I48.0,I48.1,I48.11,I48.19,I48.2,I48.20,I48.21,I48.91
Atrial flutter (AFL)	I48.3,I48.4,I48.92
End-stage renal disease (ESRD)	N18.6
Amyloidosis	E85.0,E85.1,E85.2,E85.3,E85.4,E85.8,E85.81,E85.82,E85.89,E85.9
Left ventricular-assist device (LVAD)	Z95.811
Carpal tunnel syndrome	G56.00,G56.01,G56.02,G56.03
Spinal stenosis	M48.00,M48.01,M48.02,M48.03,M48.04,M48.05,M48.06,M48.061,M48.062,M48.07, M48.08
Knee joint prosthesis	Z47.33,Z96.651,Z96.652,Z96.653,Z96.659
Hip joint prothesis	Z47.32,Z96.641,Z96.642,Z96.643,Z96.649
Nephrotic syndrome	N04.0,N04.1,N04.2,N04.3,N04.4,N04.5,N04.6,N04.7,N04.8,N04.9,N04.A
History of falls	FallHx > 0 or FallEvent >= 2 or FallNotes > 0
	FallNotes: HBPC Fall Note, Fall Note past 2 y
	FallHx: R29.6,Z91.81;
	FallEvent: W00.0XXA,W00.0XXD,W00.0XXS,W00.1XXA,W00.1XXD,W00.1XXS, W00.2XXA,W00.2XXD, W00.2XXS,W00.9XXA,W00.9XXD,W00.9XXS,W01.0XXA, W01.0XXD,W01.0XXS,W01.10XA,W01.10XD, W01.10XS,W01.110A,W01.110D, W01.110S,W01.111A,W01.111D,W01.111S,W01.118A,W01.118D, W01.118S, W01.119A,W01.119D,W01.119S,W01.190A,W01.190D,W01.190S,W01.198A, W01.198D, W01.198S,W03.XXXA,W03.XXXD,W03.XXXS,W05.0XXA,W05.0XXD, W05.0XXS,W05.1XXA,W05.1XXD, W05.1XXS,W05.2XXA,W05.2XXD,W05.2XXS,W06. XXXA,W06.XXXD,W06.XXXS,W07.XXXA,W07.XXXD, W07.XXXS,W08.XXXA,W08. XXXD,W08.XXXS,W09.0XXA,W09.0XXD,W09.0XXS,W09.1XXA,W09.1XXD, W09.1XXS, W09.2XXA,W09.2XXD,W09.2XXS,W09.8XXA,W09.8XXD,W09.8XXS,W10.0XXA, W10.0XXD, W10.0XXS,W10.1XXA,W10.1XXD,W10.1XXS,W10.2XXA,W10.2XXD, W10.2XXS,W10.8XXA,W10.8XXD, W10.8XXS,W10.9XXA,W10.9XXD,W10.9XXS,W11.

(*Continued*)

 © 2025. The Author(s).

Supplementary Table S3 (*Continued*)

Data point	Definition
	XXXA,W11.XXXD,W11.XXXS,W12.XXXA,W12.XXXD, W12.XXXS,W13.0XXA, W13.0XXD,W13.0XXS,W13.1XXA,W13.1XXD,W13.1XXS,W13.2XXA,W13.2XXD, W13.2XXS,W13.3XXA,W13.3XXD,W13.3XXS,W13.4XXA,W13.4XXD,W13.4XXS, W13.8XXA,W13.8XXD, W13.8XXS,W13.9XXA,W13.9XXD,W13.9XXS,W14.XXXA,W14. XXXD,W14.XXXS,W15.XXXA,W15.XXXD, W15.XXXS,W16.011A,W16.011D,W16.011S, W16.012A,W16.012D,W16.012S,W16.021A,W16.021D, W16.021S,W16.022A, W16.022D,W16.022S,W16.031A,W16.031D,W16.031S,W16.032A,W16.032D, W16.032S,W16.111A,W16.111D,W16.111S,W16.112A,W16.112D,W16.112S, W16.121A,W16.121D, W16.121S,W16.122A,W16.122D,W16.122S,W16.131A, W16.131D,W16.131S,W16.132A,W16.132D, W16.132S,W16.211A,W16.211D, W16.211S,W16.212A,W16.212D,W16.212S,W16.221A,W16.221D, W16.221S, W16.222A,W16.222D,W16.222S,W16.311A,W16.311D,W16.311S,W16.312A, W16.312D, W16.312S,W16.321A,W16.321D,W16.321S,W16.322A,W16.322D, W16.322S,W16.331A,W16.331D, W16.331S,W16.332A,W16.332D,W16.332S, W16.41XA,W16.41XD,W16.41XS,W16.42XA,W16.42XD, W16.42XS,W17.0XXA, W17.0XXD,W17.0XXS,W17.1XXA,W17.1XXD,W17.1XXS,W17.2XXA,W17.2XXD, W17.2XXS,W17.3XXA,W17.3XXD,W17.3XXS,W17.4XXA,W17.4XXD,W17.4XXS, W17.81XA,W17.81XD, W17.81XS,W17.82XA,W17.82XD,W17.82XS,W17.89XA, W17.89XD,W17.89XS,W18.00XA,W18.00XD, W18.00XS,W18.01XA,W18.01XD, W18.01XS,W18.02XA,W18.02XD,W18.02XS,W18.09XA,W18.09XD, W18.09XS, W18.11XA,W18.11XD,W18.11XS,W18.12XA,W18.12XD,W18.12XS,W18.2XXA, W18.2XXD, W18.2XXS,W18.30XA,W18.30XD,W18.30XS,W18.31XA,W18.31XD, W18.31XS,W18.39XA,W18.39XD, W18.39XS,W19.XXXA,W19.XXXD,W19.XXXS
Neurocardiogenic syncope (NCS)	R55.,G90.01
Biceps tendon rupture (BTR)	M66.321,M66.322,M66.329,M66.821,M66.822,M66.829
Monoclonal gammopathy of undetermined significance (MGUS)	D47.2
Other arrhythmia	I49.01,I49.02,I49.1,I49.2,I49.3,I49.40,I49.49,I49.5,I49.8,I49.9
Acute pericarditis	I30.0,I30.1,I30.8,I30.9
Acute coronary syndrome (ACS)	I20.2,I21.A1,I20.8,I20.9,I23.0,I23.5,I23.7,I24.0,I24.9,I20.0,I20.1,I21.9,I23.6,I23.8, I24.8,I21.A9,I23.1, I23.2,I23.3,I23.4,I24.1
Dysautonomia	G90.01,G90.09,G90.1,G90.2,G90.3,G90.4,G90.50,G90.511,G90.512,G90.513, G90.519,G90.521, G90.522,G90.523,G90.529,G90.59,G90.8,G90.9,G90.A
ST-elevated myocardial infarction (STEMI)	I21.01,I21.02,I21.09,I21.11,I21.19,I21.21,I21.29,I21.3,I22.0,I22.1,I22.8,I22.9
Non-ST-elevated myocardial infarction (NSTEMI)	I21.4,I22.2
Cardiology visits	Encounters in stop code 303 in the last 12 months
Cardiologist	Provider with the most encounters identified in CardioVisits
Next cardiology appointment date	Next future appointment related to Cardiology (Medicine) or Cardiology (Echo)
Next cardiology appointment location	Location of next future appointment related to Cardiology (Medicine) or Cardiology (Echo)
Next PCP appointment date	Next future appointment related to Primary Care and Community Clinics
Next PCP appointment location	Location of next future appointment related to Primary Care and Community Clinics
Last PCP visit	Any encounters (VVC, F2F, phone, telehealth) in PC stop codes in the last 12 mo
Last 2 y of admissions	Count of any inpatient admission (acute, MH, CLC, domicilliary, blind rehab, etc.) within the past 2 y
Last 2 y of ED visits	Count of ED visits within the past 2 y
History of cardiology admission	Dates of cardio admissions where the principal Dx involves CHF, CAD, MI, AFIB, CM, AVS, Arrhythmia, Acute Pericarditis, ACS)

 © 2025. The Author(s).

Supplementary Table S3 (*Continued*)

Data point	Definition
Medication history	
Diuretic	Active med in Drug Class CV70% in past 2 y
BB	Active med in Drug Class CV100 in past 2 y
ACE/ARB	Active med in Drug Class CV400,CV800,CV805 in past 2 y
Hydroxychloroquine	Active med %HYDROXYCHLOROQUINE% in past 2 y
Tafamidis	Active med %TAFAMIDIS% in past 5 y
Diflunisal	Active med %Diflunisal% in past 2 y
Patisiran	Active med %Patisiran% in past 2 y
HT	Active Home Telehealth patient
NT-BNP	LOINC: 33762-6 past 5 y
Brain natriuretic peptide (BNP)	LOINC: 30934-4 past 5 y
BNP within the Last 90 d	NT-BNP or BNP
Troponin I	TUC: TROPONIN I or POC TROPONIN (ISTAT)
	TPA: CARDIAC TROPONIN-I or POCT TROPONIN-I
	GLA: TROPONIN HIGH SENSITIVITY
Troponin within the last 90 d	
BNP or troponin flag	NT-BNP > = 900 or BNP > = 200 and Troponin I between 0.02 and 0.2 past 5 y
Creatinine	LOINC: 2160-0, 38483-4 past 5 y
Estimated glomerular filtration rate (eGFR)	LOINC: 98979-8, 33914-3, 45066-8, 48642-3, 48643-1, 62238-1, 69405-9 past 5 y
Serum protein electrophoresis (SPEP)	LOINC: 12783-7 past 5 y
Urine protein electrophoresis (UPEP)	LOINC: 34539-7 past 5 y
Kappa (K) or lambda (L) light chains (LC)	Last 5 y
Kappa LC	LOINC: 98979-8
Lambda LC	LOINC: 36916-5
K/L ratio	LOINC: 33944-0
Cocaine within the last 90 d	LOINC: 3397-7 last 90 d
Amphetamines within the last 90 d	LOINC: 3349-8 last 90 d
Weight	Weight vital signs past 5 y
EF_Slope	(nSxy – SxSy) / (nSx^2 – (Sx)^2)
EF_Slope_Avg	Average EF
EF_Slope_Last	Most recent EF
EF_SLope_N	count of EF measurements
Echo_Hx	Echocardiograms past 5 y
EchoDateTime1	Most recent echocardiogram
RWT1	septal wall thickness + posterior wall thickness divided by LV diastolic diameter. If septal WT is not provided, then posterior wall thickness × 2 divided by LV diastolic diameter
EchoDateTime2	Second to the most recent echocardiogram
RWT2	septal wall thickness + posterior wall thickness divided by LV diastolic diameter. If septal WT is not provided, then posterior wall thickness × 2 divided by LV diastolic diameter

(*Continued*)

 © 2025. The Author(s).

Supplementary Table S3 (*Continued*)

Data point	Definition
Amyloid keywords found	apical spar','cherry','amyloid','Infi% cardio'; past 5 y of echocardiogram reports (findings, conclusions)
Mayo risk score	Age + Sex + HTN + EF + PWT + RWT
Mayo risk score—age	Weight of age:
	60–69 = 2
	70–79 = 3
	>= 80 = 4
	else 0
Mayo risk score—sex	Gender is male = 2
Mayo risk score—HTN	Pt is hypertensive = -1
Average EF	If EF range is provided in echo report (e.g., 55–65), the average of the two is provided
Mayo risk score—EF	Average EF < 60 = 1
Mayo risk score—PWT	LVPWd1 >= 1.2 = 1
Mayo risk score—RWT	RWT1 > 0.57 = 2
Last TC99M	CPT Code: 78803 past 5 y
Last TAVR	CPT Code: 33361, 33362, 33363, 33364, 33365, 33366 past 14 y
Last cardiac MRI	Radiology procedures: CARDIAC MRI W W/O CONTRAST, CARDIAC MRI W/O CONTRAST past 5 y
Hospice flag	Completed hospice consult past 2 y (e-consults excluded)
ACORN screen	Completed ACORN screen past 3 y, HealthFactorType like SDOH ACORN COMPLETED%
ACORN results	List of any identified instability or insecurity issues and actions
Risk score	High risk = Davies Score >= 6 or 2 of the following factors:
	BNP: (NT-BNP > 900 or BNP > 200) and (eGFR >= 30 or null)
	Troponin: Troponin I > 0.03 or high-sensitivity Troponin > 37 for females or high-sensitivity Troponin > 57 for males
	Implanted cardiac device or pacemaker (ICD/PPM)
	Carpal tunnel syndrome (CTS)
	aFIB or atrial flutter (aFIB/AFL)
	Existence of prosthetic hip/knee joint (Hip/Knee)
	Trans-cath aortic valve replacement procedure (TAVR)
	Arrhythmia
	Acute coronary syndrome (ACS)
	Dysautonomia
	Acute pericarditis
	Nephrotic syndrome (NS)
	Neurocardiogenic syncope (NCS)
	HeartBlock
	Spinal stenosis
	FallHx
	Bicep tendon rupture (BTR)
	Monoclonal gammopathy (MGUS)
	Amyloidosis

 © 2025. The Author(s).

Supplementary Table S3 (*Continued*)

Data point	Definition
Contraindications	Any of the following conditions:
	NSTEMI
	STEMI
	ESRD
	Dialysis
	Cocaine
	Amphetamines

Supplementary Table S4 Echocardiogram keywords

Echocardiogram keywords	Apical sparing
	Cherry
	Amyloid
	Infiltrative cardiomyopathy

 © 2025. The Author(s).
